# Virome in Fecal Samples From Wild Giant Pandas (*Ailuropoda Melanoleuca*)

**DOI:** 10.3389/fvets.2021.767494

**Published:** 2021-11-12

**Authors:** Songyi Ning, Xiang Lu, Min Zhao, Xiaochun Wang, Shixing Yang, Quan Shen, Hao Wang, Wen Zhang

**Affiliations:** ^1^School of Medicine, Jiangsu University, Zhenjiang, China; ^2^Department of Clinical Laboratory, The Affiliated Huai'an Hospital of Xuzhou Medical University, Xuzhou, China

**Keywords:** wild giant panda, virome, *Genomoviridae*, *Picornavirales*, viral metagenomics

## Abstract

The giant panda (*Ailuropoda melanoleuca*) is one of the most endangered mammals in the world; anthropogenic habitat loss and poaching still threaten the survival of wild pandas. Viral infection has become one of the potential threats to the health of these animals, but the available information related to these infections is still limited. In order to detect possible vertebrate viruses, the virome in the fecal samples of seven wild giant pandas from Qinling Mountains was investigated by using the method of viral metagenomics. From the fecal virome of wild giant pandas, we determined six nearly complete genomes belonging to the order *Picornavirales*, two of which may be qualified as a novel virus family or genus. In addition, four complete genomes belonging to the *Genomoviridae* family were also fully characterized. This virological investigation has increased our understanding of the gut viral community in giant pandas. Whether these viruses detected in fecal samples can really infect giant panda needs further research.

## Introduction

The giant panda, *Ailuropoda melanoleuca*, is the only mammal belonging to the genus *Ailuropoda*, family *Ursidae*, of the order *Carnivora* (1). Giant pandas live in dense bamboo forests at an altitude of 2,500–3,500 m in southwestern China (2). In the wild, bamboo is the primary food source for giant pandas, and there are 12 general and more than 60 species of bamboo categories for giant pandas to eat. The life span of wild giant pandas is 18–20 years and can exceed 30 years in captivity. Giant pandas have lived on the earth for at least 8 million years and are the flagship species of the world's biodiversity conservation (3). By January 2021, the wild population of giant pandas in China has increased to more than 1,800.

The gut microbiome is particularly important for human health, and it is also true for giant pandas. Several studies have shown that gastrointestinal diseases are the main cause of death of giant pandas (4, 5). Some studies have pointed out that the complete transformation of giant pandas from carnivores to herbivores occurred about 2 million years ago, but they have not evolved the function of digesting celluloses and still retain a typical carnivorous digestive system (6–8). *Escherichia coli, Shigella*, and *Streptococcus* account for the vast majority of the total gut bacteria in adult pandas (9, 10), but members of *Ruminococcaceae* and *Bacteroides*, which have strong cellulose decomposing activities, have extremely low abundance in the gut of giant pandas (11). Therefore, the role of the gut microbial community in the digestion of cellulose by giant pandas remains unclear (4). With the alternation of seasons, the preference of giant pandas for bamboo parts also changes, which may even affect the composition of their gut microbial community, resulting in chronic gastrointestinal discomfort, inflammation, and mucinous feces (9). Although there have been many breakthroughs in the study of the gut microbiota of giant pandas, virological research on giant pandas has been limited to the isolation and identification of individual viruses that have developed related symptoms, such as influenza A (H1N1) and canine distemper virus (12–14).

Here, we collected seven fecal samples of wild giant pandas in Qinling Mountains and analyzed the composition of their gut viral community using the metagenomic approach. This research aims to help us explore potential vertebrate viruses from wild giant pandas and analyze their genetic relationship.

## Materials and Methods

### Sample Collection and Preparation

From May to July 2016, fresh fecal specimens of seven healthy adult wild giant pandas were collected using disposable absorbent cotton swabs in the southern slope area of the middle section of the Qinling Mountains at an altitude of about 2,500–3,000 m of Mainland China. Fecal samples were collected using disposable materials. All specimens were stored in sterilized covered containers and transported over dry ice. Prior to viral metagenomic analysis, 10 g of each fecal sample was suspended using 5 ml Dulbecco's phosphate-cushioned saline (DPBS) and vortexed for 5 min vigorously, then incubated at 4°C for 30 min. After centrifugation (10 min, 15,000 g), 1 ml of each supernatant was collected in 1.5-ml centrifuge tubes and stored at −80°C for later use (15). Since these giant pandas are wild, we do not know their exact age. Their approximate age, physical condition, and freshness of the feces were inferred by local experienced forest workers based on the characteristics of feces.

### Viral Metagenomic Analysis

A total of 500 μl of supernatant was pipetted from the seven samples (71.43 μl per sample was a pipetted from) and collected into a new 1.5-ml tube. The samples were centrifuged at 4°C for 5 min at 12,000 g and then filtered through a 0.45-μm filter (Merck Millipore, MA, USA) to remove bacterial cell-sized and eukaryotic particles. Filtrates were then treated with DNase (Turbo DNase from Ambion, Thermo Fisher, Waltham, MA, USA; Baseline-ZERO from Epicentre, Charlotte, NC, USA; and Benzonase from Novagen, Darmstadt, Germany) and RNase (Promega, Madison, WI, USA) to digest unprotected nucleic acids at 37°C for 60 min (16). Total nucleic acids were then extracted using QIAamp MinElute Virus Spin Kit (Qiagen, HQ, Germany) according to the manufacturer's protocol. These nucleic acid samples containing DNA and RNA viral sequences were subjected to reverse transcription reactions with SuperScript III reverse transcriptase (Invitrogen, Carlsbad, CA, USA) and 100 pmol of a random hexamer primer, followed by a single round of DNA synthesis using Klenow fragment polymerase (New England Biolabs, Ipswich, MA, USA). One library was constructed using the Nextera XT DNA Sample Preparation Kit (Illumina, CA, USA) and sequenced on the MiSeq Illumina platform with 250 base paired ends with dual barcoding. Paired end reads of 250 bp generated by MiSeq were debarcoded using vendor software from Illumina for bioinformatics analysis. An in-house analysis pipeline running on a 32-node Linux cluster was used to process the data. Reads were considered duplicates if bases 5–55 were identical and only one random copy of duplicates was kept. Clonal reads were removed, and low sequencing quality tails were trimmed using Phred quality score 20 as the threshold. Adaptors were trimmed according to the default parameters of VecScreen, which is the National Center for Biotechnology Information (NCBI) BLASTn with specialized parameters designed for adapter removal. The cleaned reads were *de novo* assembled within each barcode using the ENSEMBLE assembler (17). All generated contigs and singlet reads were then matched against a customized viral proteome database using BLASTx with an E-value cutoff of <10^−5^, where the virus BLASTx database was compiled using the NCBI virus reference proteome (https://ftp.ncbi.nih.gov/refseq/release/viral/) to which viral protein sequences from NCBI nr fasta file (based on annotation taxonomy in Virus Kingdom) were added. Contigs without significant BLASTx identity to the viral proteome database are searched against viral protein families in the vFam database (18) using HMMER3 to detect remote viral protein similarities (19–21).

### Acquisition and Annotation of Virus Genome

To assemble the potential viral genome of interest, the contigs which showed significant BLASTx identity to related viruses were selected in Geneious software version 11.1.2 (22). The contigs with consensus sequence length >500 bp were subjected to further analysis where the individual contig was used as reference for mapping to the raw data of its original barcode using the Low Sensitivity/Fastest parameter in Geneious 11.1.2. Those prolonged contigs with sequence length >1,500 bp and the major non-structural protein and the structural protein, as well as some contigs which only had a non-structural protein, were subjected to full characterization. Putative viral open reading frames (ORFs) were predicted by Geneious v11.1.2 with built-in parameters (minimum size: 300; genetic code: standard; start codons: ATG) (22) and were further checked through comparing to related viruses. The annotations of these ORFs were based on comparisons to the Conserved Domain Database. Putative exon and intron were predicted by Netgenes2 at http://www.cbs.dtu.dk/services/NetGene2/.

### PCR Screening

PCR screening was performed for the novel viruses of *Picornavirales* in the fecal samples to facilitate the investigation of prevalence. Sequences and characteristics of the primers used in this study are shown in Supplementary Table S2.

### Phylogenetic Analysis

To infer phylogenetic relationships, nucleotide sequences of reference strains belonging to different groups of corresponding viruses were downloaded from the NCBI GenBank database. Related nucleotide sequences were aligned using the alignment program implemented in the CLC Genomics Workbench 10.0, and the resulting alignment was further optimized using MUSCLE in MEGA v7.0 (23) and MAFFT v7.3.1 employing the E-INS-I algorithm (24). Sites containing more than 50% gaps were temporarily removed from alignments. Bayesian inference trees were then constructed using MrBayes v3.2 (25). The Markov chain was run for a maximum of 1 million generations, in which every 50 generations were sampled and the first 25% of Markov chain Monte Carlo (mcmc) samples were discarded as burn-in. Maximum likelihood trees were also constructed to confirm all the Bayesian inference trees using software MEGA v7.0 (23).

### Quality Control

Standard precautions were used for all procedures to prevent cross-sample contamination and nucleic acid degradation. Aerosol filter pipette tips were used to reduce the possibility of sample cross-contamination, and all the materials (including microcentrifuge tubes, pipette tips, etc.) which directly contacted with nucleic acid samples were RNase and DNase free. Nucleic acid samples were dissolved in DEPC-treated water, and RNase inhibitors were added. As a blank control, sterile ddH2O (Sangon, Shanghai, China) was simultaneously prepared and further processed in the same condition. During quality inspection using agarose gel electrophoresis and Agilent Bioanalyzer 2100, no detectable DNA existed in the control library. During sequencing of the Illumina MiSeq platform, the control library generated a small number of sequence reads. BLASTx searching based on the total reads in the control library revealed no viral sequences.

### Data Availability

The raw sequence reads from the metagenomic library were deposited in the Short Read Archive (SRA) of the GenBank database with accession no. SRR11348859.

All sequences obtained in this study have been deposited in the GenBank database under accession numbers MZ956587-MZ956589, MZ956591, and MZ956593-MZ956598.

## Results

### Viral Metagenomic Overview

**Figure 1:**
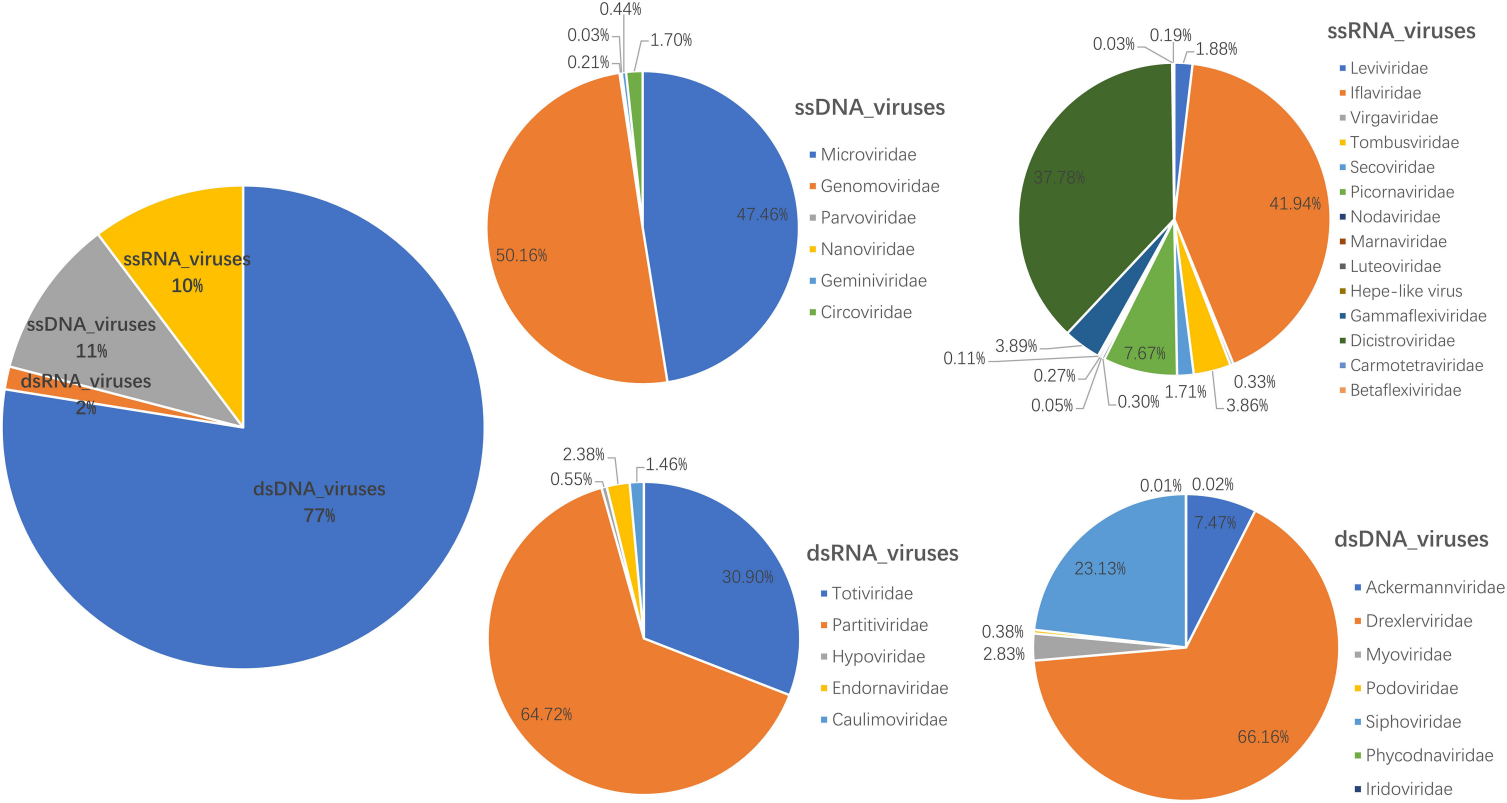
Fecal virome detected in giant pandas. The percentage of virus reads in different virus groups was shown.

The library generated a total of 393,698 raw sequence reads on the Illumina MiSeq platform; the number of unique reads after removing duplicates was 131,245. A total of 1,411 contigs were generated, and the average length of contigs was 710 bp. After bioinformatics analysis and mapping for Megan6 software visualization, a total of 35,800 sequence reads had the best matches with viral proteins (Supplementary Table S1), accounting for 9.09% of the total raw sequence reads. About 40 viral families were detected; the most abundant virus family was *Drexlerviridae* (51.29% of the total analyzed virus reads), followed by *Siphoviridae* (17.93%), *Ackermannviridae* (5.79%), *Genomoviridae* (5.35%), *Microviridae* (5.07%), *Iflaviridae* (4.31%), *Dicistroviridae* (3.88%), and *Myoviridae* (2.20%) (Figure 1 and Supplementary Table S1). In addition, there are 26,507 reads that have not been clearly assigned (E value >10^−5^), and potentially novel viruses are likely to exist in these reads.

### Identification of Novel Viruses of *Picornavirales*

**Figure 2:**
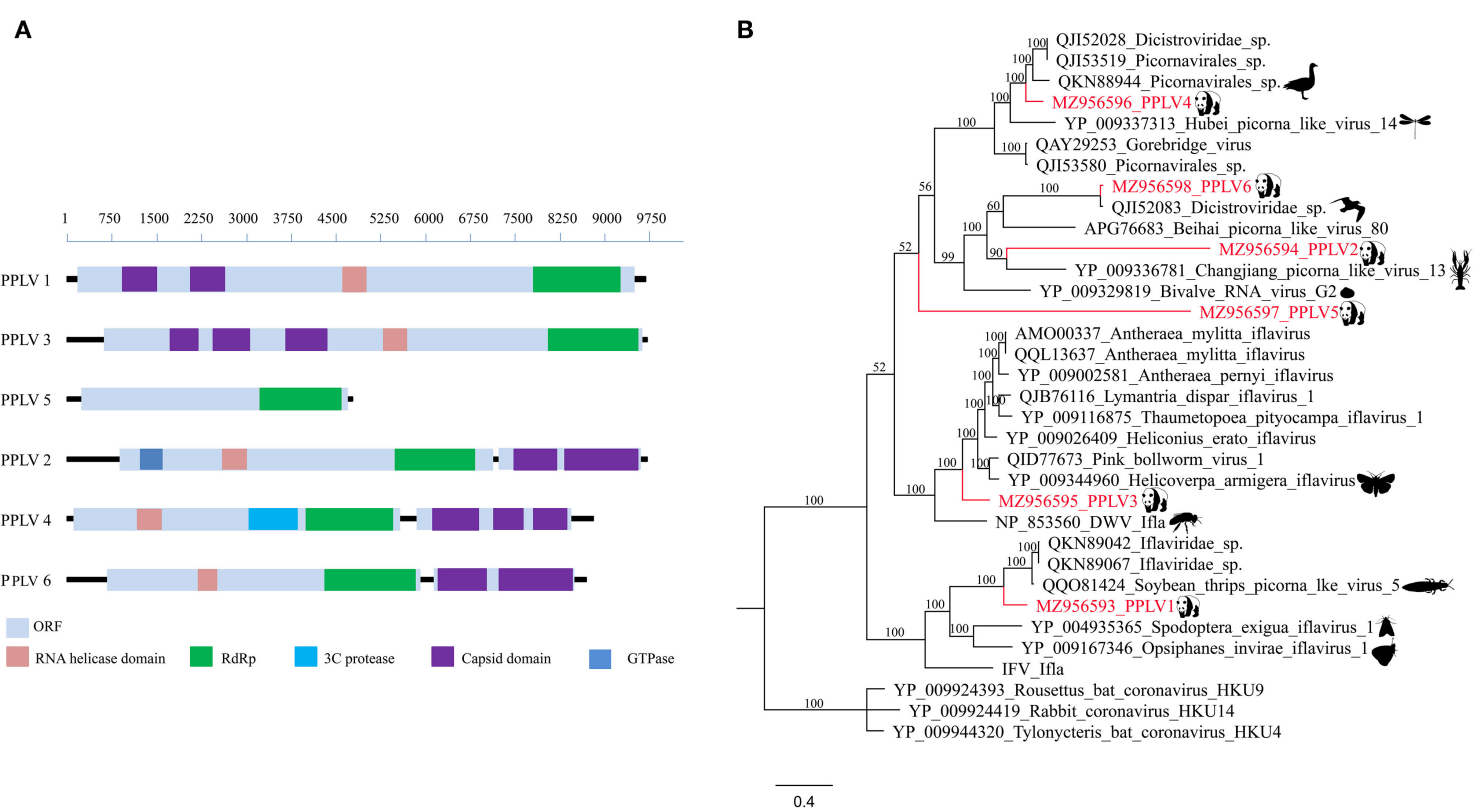
Genomic organization and phylogenetic analysis of the *Picornavirales* identified in the giant pandas. **(A)** Genomic organization of six sequences related to order *Picornavirales*. **(B)** Phylogenies of viruses belonging to *Picornavirales* identified in giant pandas. Bayesian inference tree based on amino acid sequences of RdRp of viruses belonging to *Picornavirales* identified here. Within trees, the viruses found in this study are marked with red line. Each scale bar indicates 0.4 amino acid substitutions per site.

In this study, six different RNA virus sequences (temporarily named PPLV1-PPLV6) related to *Picornavirales* were obtained using the assemble sequence program in Geneious 11.1.2; genome sizes of these six PPLVs were 9,653, 9,625, 9,670, 8,747, 4,694, and 8,638 bp, with GC contents of 41.3, 36.0, 34.3, 41.5, 34.9, and 38.3%, respectively, and their amino acid sequence identities with the best matches in GenBank ranged from 27.19 to 96.68%. The genomes of PPLV1, PPLV3, and PPLV5 all have a large ORF. PPLV1 and PPLV3 contain the Capsid domain, RNA helicase domain, and RdRp region, while PPLV5 has only one RdRp region. The genomes of PPLV2, PPLV4, and PPLV6 contain two ORFs, ORF1 and ORF2, respectively. In addition to the common RNA helicase domain, ORF1 of these three sequences also contains other different structures; for example, PPLV2 has a GTPase region, and PPLV4 has a 3C protease region, but their ORF2 all contain Capsid domain (Figure 2A). Primers were designed based on the RdRp sequence. PCR screening results showed that one sample was positive for PPLV1 and PPLV3 at the same time, and the other four viruses existed in the other four samples, respectively (Supplementary Table S2).

Phylogenetic analysis based on RdRp amino acid sequences indicated that PPLV1 clustered in the clades between the genus *Iflavirus* and Picorna-like virus, which could be considered a putative novel genus obviously. Similarly, PPLV3, as a separate branch, clustered with the genus *Iflavirus*. In addition, PPLV4, as an independent clade, was close to the Picorna-like virus. PPLV6 was clustered with the previously reported unclassified Dicistroviridae identified in the bird's intestines and has a 96.68% amino acid sequence identity. PPLV2 and PPLV5 shared 30.92 and 27.19% amino acid sequence identity with the best matches in the GenBank database, respectively. Furthermore, PPLV5 was classified outside the currently well-defined families, which makes it possible for the existence of a novel virus family (Figure 2B).

### Four Viruses Belonging to the Family *Genomoviridae*

**Figure 3:**
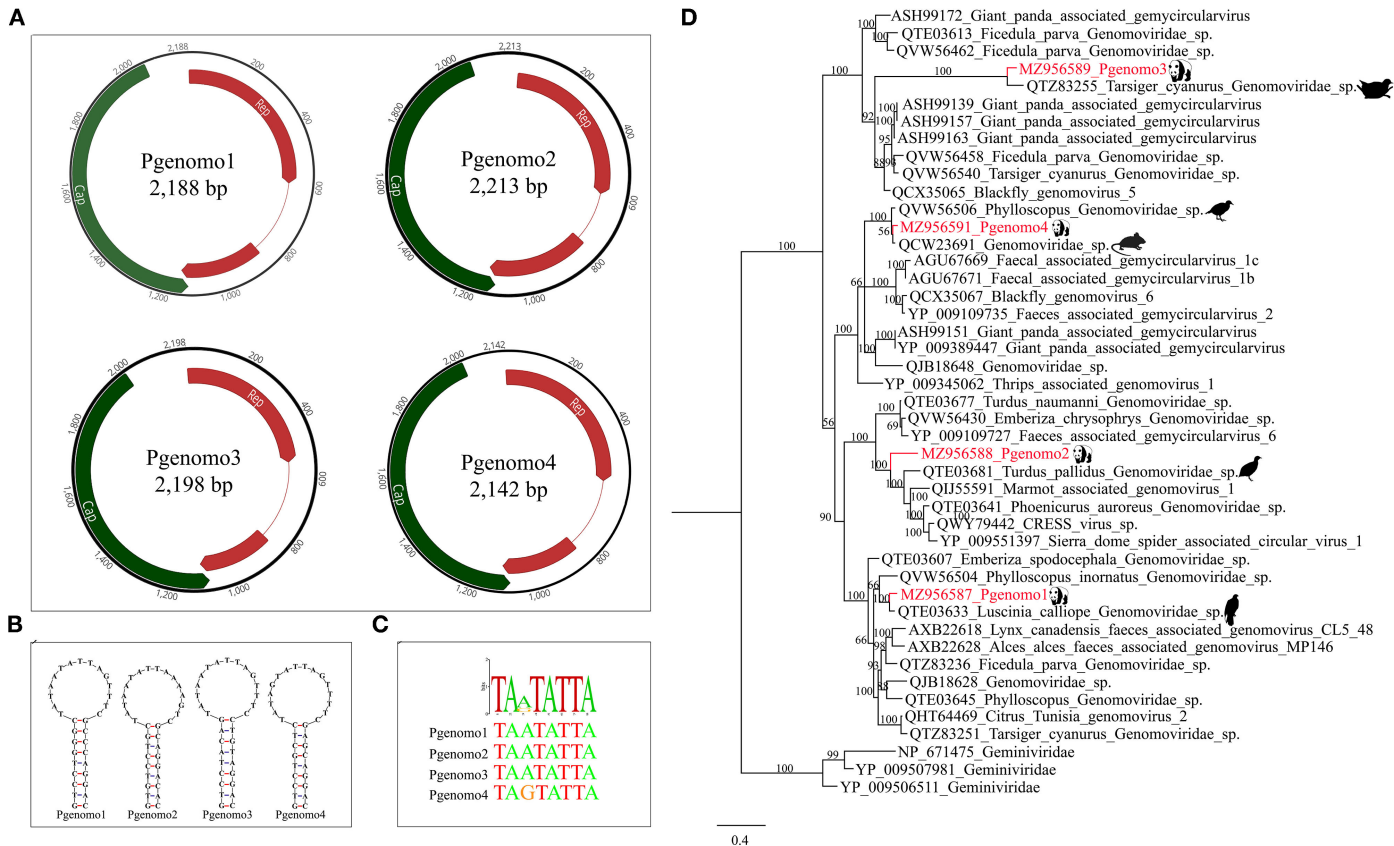
Phylogenetic analysis and genomic organization of the family *Genomoviridae* in the giant pandas. **(A)** Genomic organization of four sequences related to family *Genomoviridae*. **(B)** The stem-loop structures of the four genomes identified in giant pandas. **(C)** The non-amer in the stem-loop structure of the four genomes identified in giant pandas. **(D)** Phylogenies of viruses belonging to family *Genomoviridae* identified in giant pandas. Bayesian inference tree based on amino acid sequences of Rep of viruses belonging to family *Genomoviridae* identified here. Within trees, the viruses found in this study are marked with red line. Each scale bar indicates 0.4 amino acid substitutions per site.

In this study, we have identified four complete genomovirus-related sequences through assembly and mapping, namely, Pgenomo1-4. The whole genome sizes of Pgenomo1-4 were 2,188, 2,213, 2,198, and 2,142 bp, respectively (Figure 3A). The stem-loop structure was found between the 5′ ends of the Rep protein and Cap protein of the genomes of all the four viruses (Figure 3B), all of which contained a conserved non-amer of “TARTRTTK” (Figure 3C). BLASTp searching in the GenBank database with the predicted Rep protein sequence indicated that the best match of Pgenomo1 was Luscinia calliope Genomoviridae sp. (QTE03633) from wild bird feces, sharing 90.69% amino acid sequence identity. The Rep protein of Pgenomo2 was closely related to Turdus pallidus Genomoviridae sp. (QTE03681), sharing 67.92% amino acid identity with it, but the genome-wide nucleotide identity was 81.70%; based on the genome-wide pairwise sequence identity threshold of 78%, this virus cannot be considered as a new species (26). The amino acid sequence identity between Pgenomo3 and its best matching Tarsiger cyanurus Genomoviridae sp. (QTZ83255) was 81.56%. Similarly, the best matches of Pgenomo4 was Phylloscopus Genomoviridae sp. (QVW56506) from wild birds, sharing 91.32% amino acid sequence identity (Figure 3D).

## Discussion

The giant panda is regarded as the “living fossil” and “China's national treasure,” the image ambassador of the Worldwide Fund for Nature, and the flagship species of the world's biodiversity conservation (27, 28). A great deal of effort has been put into preserving this iconic species; increasing evidence emphasizes the close interaction between the gut microflora and animal health (29, 30). However, so far, only a limited number of studies have explored the gut virome of wild giant pandas. In this study, we investigated the gut virome from seven wild giant pandas; overall, viruses that may infect bacteriophages, vertebrates, plants, and insects have been detected (Supplementary Table S1), and the number of bacteriophages accounted for the majority of the total number of viruses, with families *Drexlerviridae* and *Siphoviridae* (infected with *Shigella* species or *E. coli*) (31, 32), overwhelmingly accounting for ~51.29 and 17.93%, respectively. However, this is different from the results reported by the previous study that families *Myoviridae* (46.22%), *Siphoviridae* (23.98%), and *Podoviridae* (8.78%) were dominant in the feces of adult giant pandas (33). This may be due to the limited sample size and the lack of construction of libraries according to the age difference of giant pandas. Genomovirus, which can infect humans, mammals, or birds, and Iflavirus, which can infect insects, accounted for only a small part of the total virome, and even some other picornaviruses that can infect vertebrates can only be detected by the method of metagenomics sequence assembly and mapping. Compared with other mammals, the gut viral community diversity of wild pandas is a bit low (9), which may be related to the low amount of fecal content analyzed in this study. In our previous report (14), the library numbered 16FNW (164,066 unique reads) contained only one wild giant panda, and the Shannon index calculated based on the viral family had a higher alpha diversity than the library constructed by seven wild giant pandas in this study (2.36:1.29), Therefore, it may be appropriate to construct one library of all fecal samples together. In addition, that article has a larger sample size and diverse sample types (feces, blood, nasopharyngeal secretions, and tissues), so relatively more viruses have been detected. In addition, this may also be related to the single food source of wild pandas, and whether this phenomenon is related to the endangered extinction of this species in the past remains to be studied. Several documents have pointed out that in different seasons, pandas' different preferences for bamboo parts may cause differences in their gut microbial communities (9, 34, 35), and it is not known whether this will affect the composition of the gut virome. It is a pity that we did not try to collect samples based on seasonal changes as a control to understand this.

The polyprotein encoded by order *Picornavirales* generally contains the RNA helicase domain, RNA-dependent RNA polymerase (RdRp), 3C protease, Capsid domain, and GTPase. The genomic nucleotide sequences of different *Picornavirales* members have a high degree of divergence, and their genomic organization patterns are also very different between different families (36). Although many recent studies have identified more members of *Picornavirales*, the interaction between *Picornavirales* and their diseases is still unclear (37, 38). It seems that any research on viral metagenomics can find viral sequences that have never been described in various samples of animal or human; these sequences are called “viral dark matter” (39); this is the advantage of metagenomics. Although the diversity of giant pandas' gut virome was lower than that of other mammals, the characterization of some viruses seems to define novel viral families, genera, or species, just as genomes identified through unbiased high-throughput sequencing analysis in this study contributed several new evolutionary branches to *Picornavirales* (Figure 2B). The PPLV3 and PPLV6 identified in this study were closely related to dicistrovirus and iflavirus. Dicistrovirus and iflavirus mainly infect invertebrates or insects (40, 41). According to phylogenetic analysis, PPLV3 may be derived from *Helicoverpa armigera* or *Apis mellifera*, and PPLV6 may be derived from bird excrement. In the past few years, viruses from the family *Genomoviridae* have been frequently found to be associated with a variety of samples ranging from fungi to animal sera, indicating that genomoviruses are widespread and abundant in the environment, but their true host range is still unknown. Although the Cap protein of genomovirus has no obvious similarity with other known viruses at the sequence level, its Rep protein is homologous to other eukaryotic single-stranded (ss) DNA viruses and has the highest similarity with the family *Geminiviridae*, sharing special sequence motifs and forming a sister group in phylogenetic analysis (42). In this study, the virus strains that were closest to the four Genomoviridae-related genomes identified from panda fecal samples were all isolated from bird feces. It should be noted here that some genomes described in this study share high homology with viruses previously described in birds. This may be due to the fact that these viruses belonging to *picornavirales* or *genomoviridae* are widely present in environments around the world (such as insects or plants that are eaten by birds and pandas) (43–45). In addition, these viruses described in birds were derived from feces rather than tissues or blood, so it is still unknown whether these viruses can replicate in the bird's body. In conclusion, this study summarizes an overview of the fecal virome of wild giant pandas in the south slope of the middle section of Qinling Mountains, provides useful information for monitoring the health of these animals, and helps provide a valuable baseline for the prevention and treatment of possible future viral diseases of giant pandas in this region.

In the virome of giant panda fecal samples, we also found a small number of sequence reads showing significant sequence identity to Hepe-like virus and papillomavirus. The Hepe-like virus sequences had the best matches (with sequence identity of 50%) to Hepe-like viruses from mollusk, suggesting that these sequences were derived from giant pandas' diet. These papillomavirus sequences showed high sequence identity (100%) to *Rattus norvegicus papillomavirus 2*, suggesting that the papillomavirus sequences were also from diet as wild giant panda can feed on wild rats.

## Data Availability Statement

The datasets presented in this study can be found in online repositories. The names of the repository/repositories and accession number(s) can be found in the article/Supplementary Material.

## Ethics Statement

The animal study was reviewed and approved by Jiangsu University Ethics Committee. Written informed consent was obtained from the owners for the participation of their animals in this study.

## Author Contributions

XW, SY, QS, HW, and WZ conceived and designed the study. SN, XL, MZ, and HW carried out the study. SN, XL, MZ, XW, and HW analyzed the data. SN and XL contributed to writing the manuscript. All authors have read and approved the final manuscript.

## Funding

This work was supported by the National Key Research and Development Programs of China for Virome in Important Wildlife (2017YFC1200201), Chengdu Research Base of Giant Panda Breeding (2020CPB-C11), and Donghai People's Hospital-School of Medicine UJS Joint Laboratory Foundation (20210467).

## Conflict of Interest

The authors declare that the research was conducted in the absence of any commercial or financial relationships that could be construed as a potential conflict of interest.

## Publisher's Note

All claims expressed in this article are solely those of the authors and do not necessarily represent those of their affiliated organizations, or those of the publisher, the editors and the reviewers. Any product that may be evaluated in this article, or claim that may be made by its manufacturer, is not guaranteed or endorsed by the publisher.

## Supplementary Material

Supplementary Table S1 The summary of library information of giant pandas.

Supplementary Table S2 Primers used for specific PCR confirmation.
