# Supplementary material for: Virome in Fecal Samples From Wild Giant Pandas (Ailuropoda Melanoleuca)
Source: Front Vet Sci. 2021 Nov 12;8:767494. doi: 10.3389/fvets.2021.767494 (PMC8636094; doi:10.3389/fvets.2021.767494)
Supplement: Supplementary Table S2 — Primers used for specific PCR confirmation. [file Table_2.DOCX]

**Supplementary Table 2. Primers used for specific PCR confirmation.**

| **Primer** | **Targeted virus** | **Sequence (5´-3´)** | **Fragment size (bp)** | **Annealing temperature (^o^C)** |
| --- | --- | --- | --- | --- |
| **PPLV1 sense** | **PPLV1** | **TGGTTAGTTCCCGTATGGCG** | **723** | **59** |
| **PPLV1 antisense** |  | **GCACATCAAGAGCCGATTGC** |  |  |
| **PPLV2 sense** | **PPLV2** | **ACTCTGGCCGTGATGCTTAG** | **836** | **58** |
| **PPLV2 antisense** |  | **ACGGGTGGTGCATTAATCTC** |  |  |
| **PPLV3 sense** | **PPLV3** | **TGAGAGGAATGCATGGAGAG** | **822** | **59** |
| **PPLV3 antisense** |  | **CACAACTAAGACGAGGGTGC** |  |  |
| **PPLV4 sense** | **PPLV4** | **CTGCGCCAATGCATTACGTC** | **888** | **59** |
| **PPLV4 antisense** |  | **GGTGAAGTGCTAATTCGCGC** |  |  |
| **PPLV5 sense** | **PPLV5** | **GACTCGCTACAACATGTGTG** | **619** | **56** |
| **PPLV5 antisense** |  | **CTGATATCTGGCTCGTAATG** |  |  |
| **PPLV6 sense** | **PPLV6** | **CTGGTATAGAGTTCTGTGAG** | **1114** | **59** |
| **PPLV6 antisense** |  | **GGTGAGTGGAGCGACAATTC** |  |  |
